# A Rare Case of Primary Sarcoma Arising Within Free Muscle Transfer

**DOI:** 10.1055/a-2303-4940

**Published:** 2024-06-10

**Authors:** Anchal Jain, Bilal Rafique, Amer J. Durrani, Ahid Abood

**Affiliations:** 1 Department of Plastic and Reconstructive Surgery, Addenbrooke's Hospital, Cambridge University Hospitals NHS Trust, Cambridge, United Kingdom

**Keywords:** sarcoma, lower limb trauma, reconstruction, flap, wound healing

## Abstract

Soft tissue sarcomas are one of the rarest forms of cancer. We describe a unique case of a 35-year-old patient who sustained an open lower limb fracture requiring an intramedullary nail and free latissimus dorsi (LD) muscle flap reconstruction. He had a complex postoperative course including osteomyelitis, a refracture, and chronic pain. Eleven years following the injury, he presented with pain and localized swelling around the flap. Histological analysis confirmed a rhabdomyosarcoma within the LD muscle and he underwent a transfemoral amputation. He is now in remission and walks on a prosthesis pain-free.

## Introduction

Muscle and fasciocutaneous flaps are common reconstructive options for patients with traumatic lower limb soft tissue defects. The latissimus dorsi (LD) is one of the most commonly used muscles for coverage of such defects. It lends itself well to this purpose by offering a robust, large, and well-vascularized muscle with a long pedicle length and the option of a skin paddle. Alternatively, a split thickness skin graft can be applied to resurface the muscle.

Soft tissue sarcomas (STSs) are rare forms of cancer with an incidence, in England, of 7.7 cases per 100,000 persons and approximately 4,300 new cases in England each year. 1 Rhabdomyosarcoma (RMS) is a rare primitive mesenchymal type of STS made up of cells that differentiate into skeletal or striated muscle. It is the most common soft tissue malignancy in children but exceedingly rare in adults. 2

The following case highlights a sinister presentation of an RMS following a complex lower limb reconstruction. To our knowledge, only one other case report exists in the literature demonstrating a similar tumor within a muscle flap. 3 Our case report highlights how this rare tumor may present, particularly within the context of a complex postoperative course, other variables, and more likely diagnoses. It hence emphasizes this crucial but rare differential diagnosis.

## Case

**Fig. 1:**
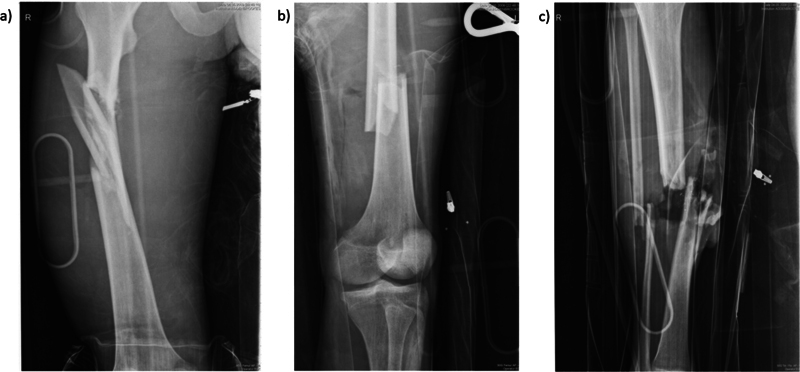
Radiographs showing ( **a** ) comminuted fracture of right femur, ( **b** ) fracture of left femur, ( **c** ) fracture of right tibia and fibula.

A 35-year-old male was involved in a high-speed road traffic accident 13 years ago. He sustained a closed fracture of the right femur and an open fracture of the left femur and right tibia and fibula with an overlying soft tissue defect (Gustilo and Anderson IIIb; Fig. 1 ).

**Fig. 2:**
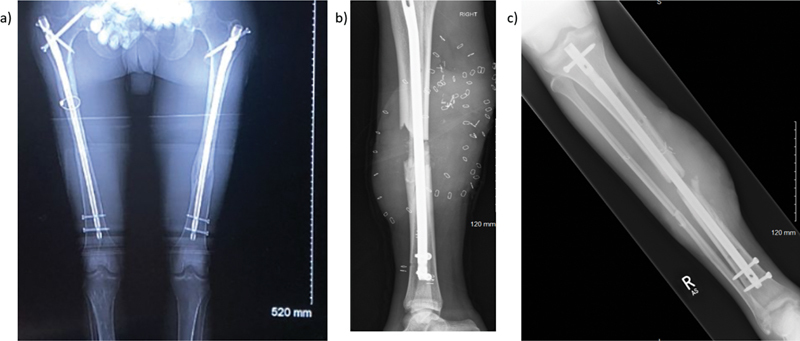
Radiographs showing ( **a** ) bilateral femur intramedullary (IM) nails, ( **b** ) IM nail fixation of right tibia and skin staples used for flap reconstruction, ( **c** ) 1 month postinjury follow-up X-ray.

**Fig. 3:**
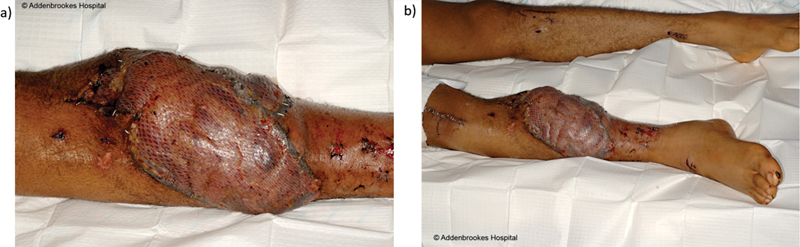
Free latissimus dorsi flap and a split skin graft reconstruction day 5 postinjury.

The bilateral femoral fractures were treated with intramedullary (IM) nails ( Fig. 2a ). The right leg underwent a joint orthoplastic debridement and external fixation on day of admission. On day 5, the external fixator was replaced with an IM nail and the soft tissue defect was reconstructed using a free LD flap and split skin graft ( Figs. 2b, c and 3 ). He was discharged 4 weeks following initial admission.

**Fig. 4:**
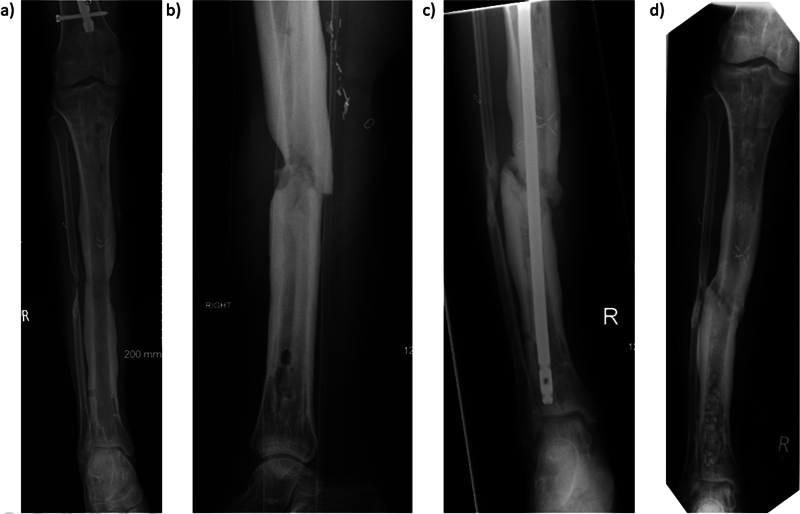
( **a** ) IM nail removed due to osteomyelitis, ( **b** ) refracture of tibia, ( **c** ) new nail inserted, ( **d** ) nail removed due to chronic pain. IM, intramedullary.

Over the course of the next few years, he had various hospital admissions due to osteomyelitis (OM), a refracture following trauma and chronic pain ( Fig. 4 ). Three months postoperatively, he developed OM of the right tibia which was treated within the bone infection unit with removal of IM nail and IV antibiotics. Three years postoperatively, he sustained trauma to the leg causing a refracture and had an IM nail reinserted. He continued to have chronic pain in the limb which was maximally treated with analgesics but eventually underwent removal of his second IM nail.

**Fig. 5:**
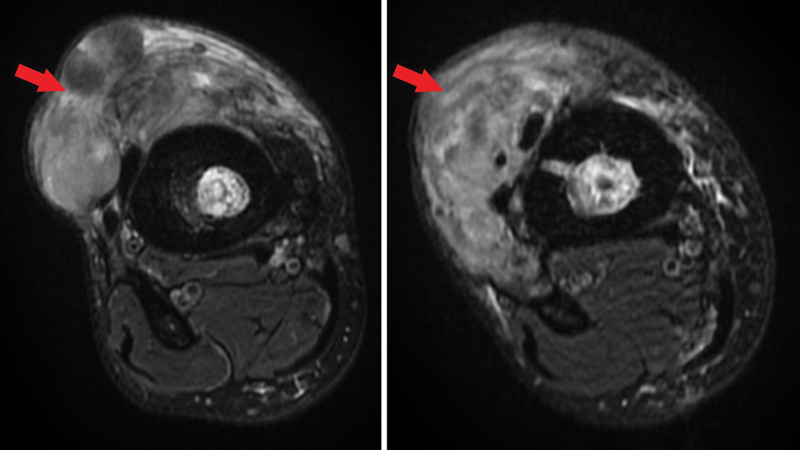
MRI revealed a large, heterogeneous (red arrow), lobulated mass anterior to the mid/distal third tibia likely an inflammatory/infective phlegmon, however, biopsy suggested to rule out neoplastic changes.

Eleven years following the initial injury, he presented with swelling and pain. This was most likely thought to be a recurrence of OM and he was managed with prolonged course of antibiotics. An MRI revealed a large inflammatory phlegmon most in keeping with infection, however a biopsy was advised to rule out neoplastic changes ( Fig. 5 ). He progressed to have a PET-CT which showed low-grade uptake in soft tissue of right anterior lower leg and confirmed no evidence of metastatic disease. A biopsy of the muscle flap revealed a spindle cell/sclerosing RMS.

**Fig. 6:**
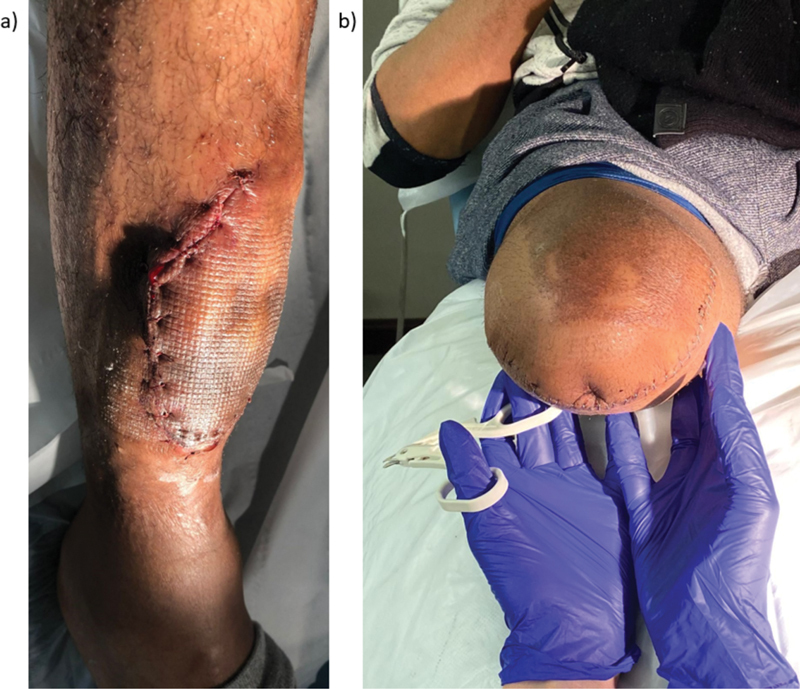
Photograph showing ( **a** ) swelling 11 years postinitial reconstruction and ( **b** ) healthy stump post-above knee amputation.

He was treated in a regional sarcoma center in-line with United Kingdom guidelines on management of STSs. 4 There was no evidence of distant metastatic disease and he was treated with the standard IVADo chemotherapy regimen (ifosfamide, vincristine, doxorubicin, dactinomycin) followed by a right above knee (transfemoral) amputation ( Fig. 6 ).

**Fig. 7:**
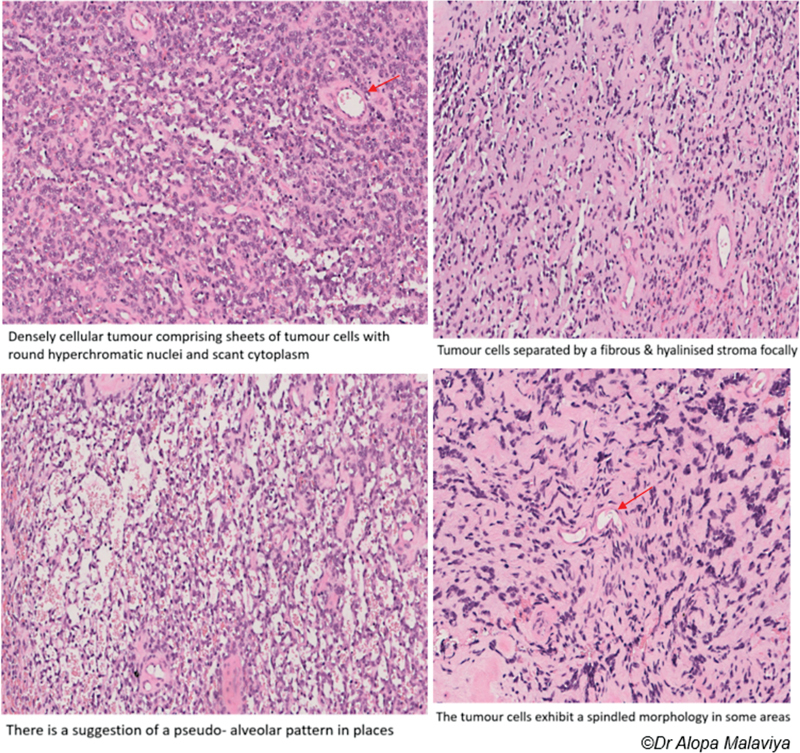
Histopathology (Hematoxylin and Eosin stain) showing features of rhabdomyosarcoma (annotated using red arrow).

Histopathology showed densely cellular tumor comprising sheets of tumor cells with round hyperchromatic nuclei and scant cytoplasm. Tumor cells exhibited a spindled morphology in some areas ( Fig. 7 ). On immunohistochemistry, the tumor showed strong diffuse staining for Desmin and Myo-D1 in keeping with the final diagnosis of pT2, N0, M0, Grade 3 sclerosing RMS (100 × 40 × 20 mm).

**Fig. 8:**
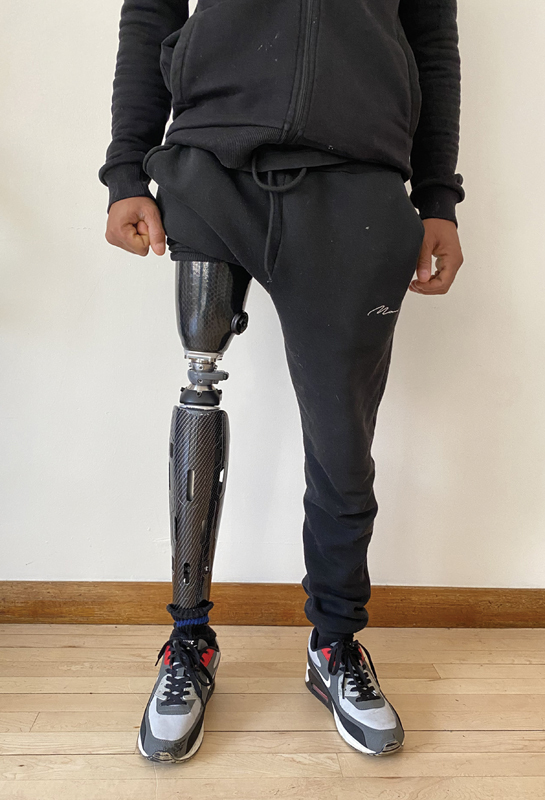
Patient weight bearing in prosthesis.

On regular clinic reviews, he continued to show positive progression with phantom limb pain which eventually settled. Surveillance staging scans showed no evidence of recurrence or metastases. He continues to be seen by specialist amputee therapy services and is fully independent walking with a prosthesis and is pain free ( Fig. 8 ).

## Discussion

STSs are rare in adults making up less than 1% of all adult malignancies. Of all STSs, RMS accounts for 3% of the cases. 5 The latest Cancer Research UK data show 4,300 new cases in England each year with an average 10-year survival rate of 45%. Many of these cases present with hematogenous metastatic disease. 1 The mainstay of treatment in adults with STS is generally adjuvant or neoadjuvant chemotherapy, surgery, and radiotherapy.

Our patient interestingly presented with a primary RMS arising within the LD free muscle flap 11 years after it was transferred to his right leg. This was on a background of significant traumatic injury followed by a prolonged course of OM, repeat trauma and surgery, and chronic pain. One of the key learning points is that this may theoretically occur within any type of soft tissue transfer. The other is within this patient's presentation. Eleven years later when he represented with pain and swelling, the most likely, and possibly concurrent, diagnosis was OM as demonstrated by the MRI. On exploration and bone biopsy it is therefore prudent to not only send samples for microbiology but also histopathological analysis. Without this, it is likely the diagnosis and potentially prognosis would have been delayed.

It is difficult to pinpoint the exact cause of this sarcoma and whether or not this may have occurred still if the muscle was not transferred. Most cases of sarcoma arise de novo, however risk factors include family or genetic history (RMS may occur in patients with Gorlin syndrome), exposure to toxins such as vinyl chloride or arsenic, radiotherapy, or HIV. Our patient had no history of risk factors. A possible theory in this case may include a sustained inflammatory response due to chronic infection and repeated trauma.

Within the context of lower limb salvage and rehabilitation, this case demonstrates very well that limb amputation should also be considered, in select cases, as a good surgical procedure and not failure. Our patient following this has been able to integrate well back into society, has returned to work, and is now pain-free walking well on a prosthesis.
